# Radiological Classification of Glenoid Deformity in Rheumatoid Arthritis

**DOI:** 10.1155/2011/239894

**Published:** 2011-10-20

**Authors:** Naoki Miyoshi, Naoki Suenaga, Kou Katayama, Naomi Oizumi, Hiroshi Yamaguchi, Takeo Matsuno

**Affiliations:** ^1^Department of Orthopedic Surgery, Asahikawa Medical College, Asahikawa 078-8510, Japan; ^2^The Upper Extremity Center of Joint Replacement and Endoscopic Surgery, Hokushin Orthopedic Hospital, Sapporo 003-0823, Japan; ^3^Katayama Orthopedic Rheumatology Clinic, Asahikawa 078-8243, Japan; ^4^Department of Orthopedic Surgery, School of Medicine, University of Ryukyus, Okinawa 903-0215, Japan

## Abstract

We report a classification system based on the changes in shape of the glenoid fossa and on an evaluation of the upward migration of the humeral head, because a simple classification based on X-ray evaluation would be of great assistance to physicians dealing with the diagnosis and treatment of RA. We evaluated 150 shoulders of 118 RA patients who showed changes in the glenoid fossa after radiological examinations. The morphology of the glenoid fossa of the RA shoulder was classified into 3 types and we were able to classify a total of six types of deformities by adding the problem of upward migration of the humeral head. An additional investigation on the difference in the type of deformity between the right and left shoulder, the changes in type during the course of the study, and the relationship between this particular classification and certain patient characteristics was also included.

## 1. Introduction

There are many studies evaluating the changes in the shape of the glenoid fossa of the scapula in patients with osteoarthritis of the shoulder [1–7], but few discuss the changes in the shape of the glenoid fossa in patients with rheumatoid arthritis (RA) [8–10]. In recent years, biologics and immunosuppressants have increasingly become the drugs of choice for treatment of RA, and these are drugs with serious side effects. Conservative treatment consisting mainly of drugs administered by rheumatologists and physicians outside the field of orthopedic surgery is another approach for treatment of RA. A simple classification based on X-ray evaluation would be of great assistance to physicians dealing with the diagnosis and treatment of RA, especially to those physicians and rheumatologists who are unfamiliar with radiological evaluation of the shoulder joint. The aims of this study were to evaluate the characteristic changes in shape of the glenoid fossa of RA patients using radiological examinations in the coronal plane and to report a classification system based on the changes in shape of the glenoid fossa and on an evaluation of the upward migration of the humeral head.

## 2. Materials and Methods

**Figure 1:**
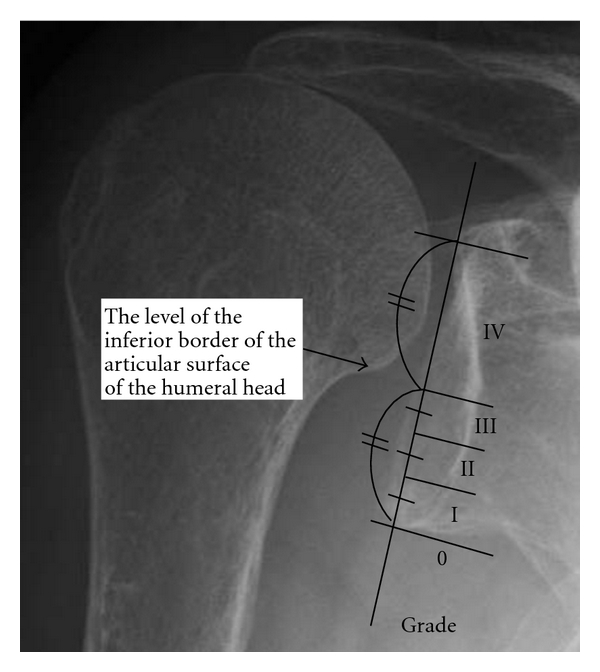
Oizumi classification. Grading of upper migration of humeral head. The inferior half of the glenoid is divided into 3 zones. The grade is defined by the position of the inferior border of the articular surface of the humeral head in the following zones: grade 0, the inferior border of the articular surface of the humeral head is below the lower glenoid rim; grades I, II, and III, the inferior border of the articular surface of the humeral head is in each zone; grade IV, the inferior border of the articular surface of the humeral head is above zone III.

**Figure 2:**
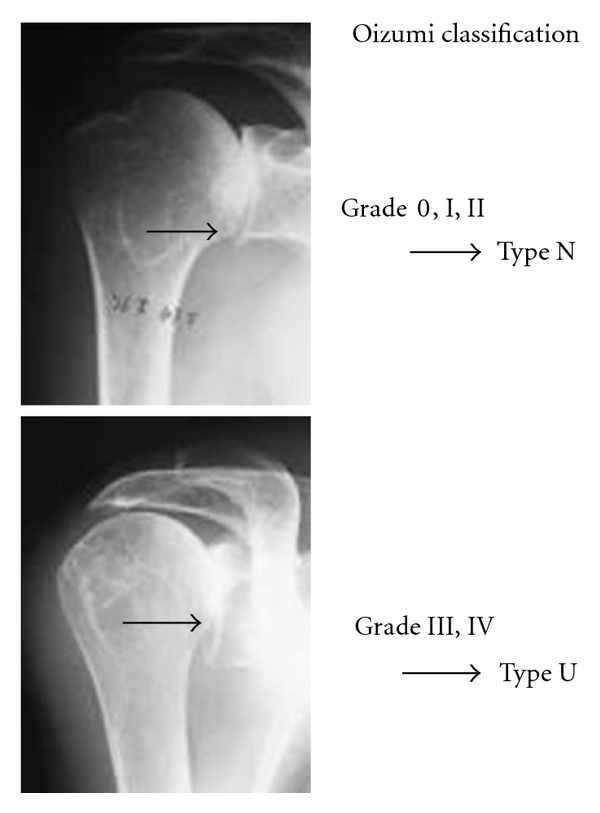


150 shoulders of 118 RA patients who showed changes in the glenoid fossa after radiological examinations were included in this study. There were 22 men (31 shoulders) and 96 women (119 shoulders), aged 21–81 years (average age: 61.3 years). The glenoid fossa deformities on the basis of the most recent anteroposterior (A-P) X-ray scans were evaluated. And also, an evaluation of the existence of upward migration of the humeral head was included in the previous classification. In the classification provided by Oizumi et al. [11] (Figure 1), those with grade III or higher upward migration of the humeral head were classified as Type U and the grades 0, I, and II were as Type N (Figure 2).

The difference in the type of deformity between the right and left shoulder and the changes in type during the course of the study for cases which could be observed for 2 years or more were also investigated.

Furthermore, the correlation with this particular classification and certain patient characteristics were also studied. The evaluated patient characteristics were age, duration of illness, use of a cane or wheelchair, Steinbrocker functional classification (class) [12] (Table 1), history of leg surgery, range of motion (flexion, external rotation, etc.), Japanese Orthopedic Association shoulder score (JOA score), existence of rotator cuff problems in cases which had an MRI, and the availability of surgery findings in cases which had undergone surgery. 

Analysis of variance (ANOVA) Fisher's protected least significant difference (PLSD) test was used for a statistical analysis, and a risk of 5% or lower was taken to be a significant difference.

## 3. Results

**Figure 3:**
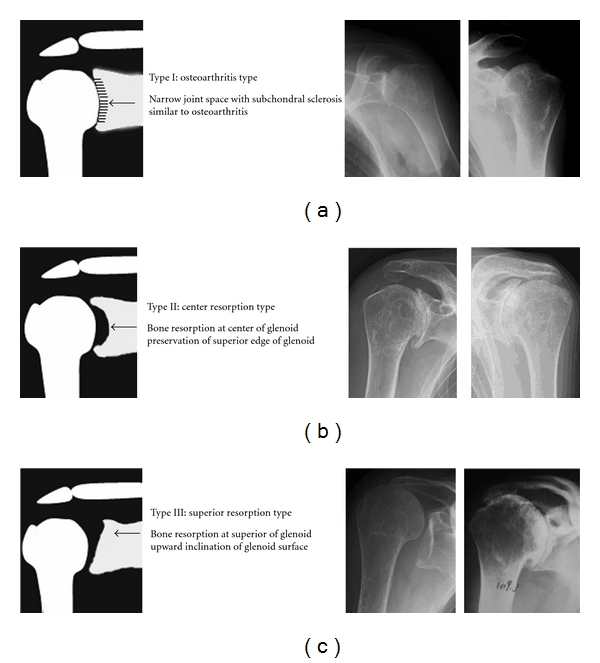
The characteristic changes in shape of the glenoid fossa of RA patients using radiological examinations in the coronal plane.

The morphology of the glenoid fossa in patients with RA of the shoulder was classified into 3 types (Figure 3). 

Type I was characterized mainly by arthritis-like changes such as loss of joint space and signs of osteosclerosis of the joint surface. There were 45 shoulders in 37 patients in this group. Type II was characterized by absorptive changes occurring at the center of the glenoid fossa but with a residual fossa upper margin. There were 57 shoulders in 43 patients in this group. Type III was characterized by absorptive changes at the upper margin of the glenoid fossa with an upward slant to the fossa. There were 48 shoulders in 38 subjects in this group.

**Figure 4:**
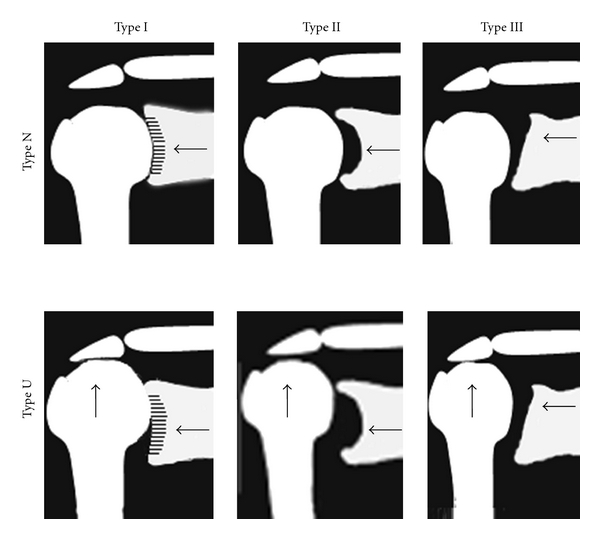
A classification system based on the changes in shape of the glenoid fossa and on an evaluation of the upward migration of the humeral head.

When upward migration of the humeral head was added to the list of changes, 36 shoulders in 29 patients (5 shoulders from 4 men, 31 shoulders from 25 women), aged 33–77 years (average age: 60.6 years), were classified as Type I-N, and 9 shoulders in 8 patients (4 shoulders in 3 men, 5 shoulders in 5 women), aged 55–75 years (average age: 67.3 years), were classified as Type I-U. In Type II, there were no cases of Type U; all were classified as Type II-N, consisting of 57 shoulders in 43 patients (12 shoulders in 7 men, 45 shoulders in 36 women), aged 21–80 years (average age: 61.7 years). Sixteen shoulders in 14 patients (2 shoulders in 2 men, 14 shoulders in 12 women), aged 25–81 years (average age: 62.5 years), were classified as Type III-N and 32 shoulders in 24 patients (8 shoulders in 6 men, 24 shoulders in 18 women), aged 50–78 years (average age: 61.8 years), were classified as Type III-U (Figure 4, Table 2). 

**Figure 5:**
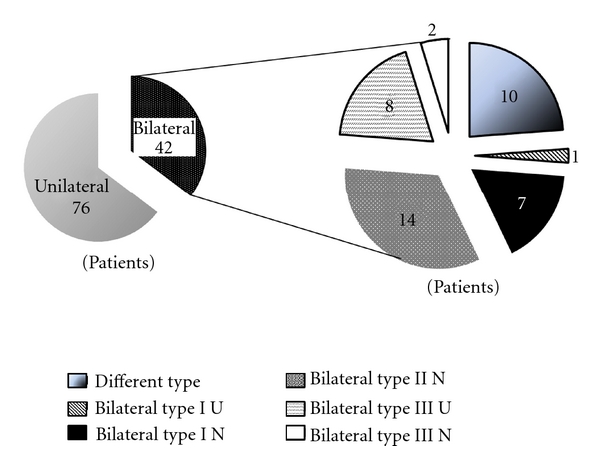
The difference in the type of deformity between the right and left shoulder in patients with deformity of both shoulders.

Of the 42 patients in which both shoulders were involved, both shoulders belonged to the same group in 32 patients (Type I-U: 1, Type I-N: 7, Type II-N: 14, Type III-U: 8, and Type III-N: 2) (Figure 5).

**Figure 6:**
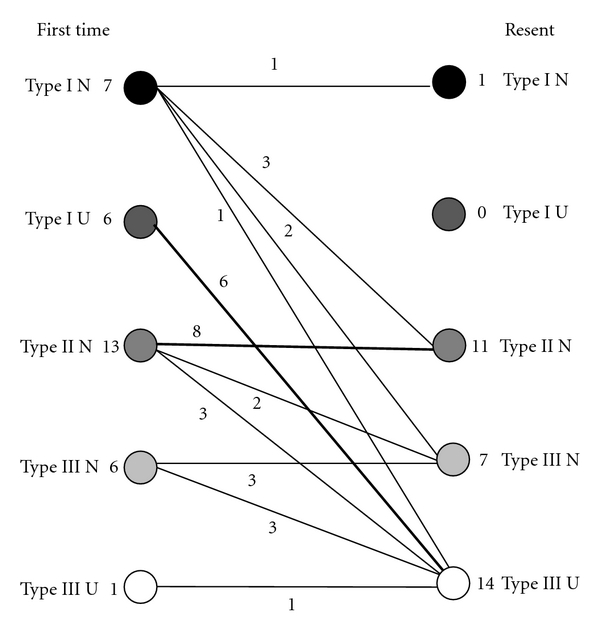
The changes in type during the course of the study for patients who could be observed for 2 years or more.

Of the 33 shoulders that could be monitored for 2 years or more, 20 shoulders changed type during that time. Of the 7 Type I-N shoulders, 3 changed to Type II-N, 2 changed to Type III-N, and 1 changed to Type III-U. In Type I-U, all 6 shoulders changed to Type III-U. Of the 13 Type II-N shoulders, 2 changed to Type III-N and 3 changed to Type III-U. Of the 6 Type III-N shoulders, 3 changed to Type III-U. The 1 Type III-U shoulder did not change (Figure 6).

In the study, when we focused on the patient characteristics such as the use of a cane or wheelchair and the Steinbrocker functional class, we found a significantly higher number classified as Type III-U. A history of leg surgery tended to be more common in Types III-U and -N than in Type II, although the difference was not significant. The range of motion in flexion and external rotation was greater in Types I-N and II-N than in Types I-U and III-U. More cases of Type I-U had rotator cuff problems than was the cases in Type I-N and II-N (Table 3).

## 4. Discussion

Previous studies on classification of RA in the shoulder have often used the Larsen classification to grade the degree to which joint destruction has advanced. On the basis of the mode of joint destruction, Neer II classified the pattern of RA shoulder structural destruction into 3 types: wet, dry, and resorptive [8]. Hirooka et al. also classified the pattern of RA shoulder structural destruction into 5 types, including non-progressive, arthrosis-like, erosive, collapse, and mutilating-type patterns, on the basis of a study on the natural course of 166 RA shoulders from 83 subjects [9]. They further describe a classification system that in addition to serving as a prognostic indicator, makes it possible to evaluate the characteristics of the degree of severity and mode of destruction. Lévigne and Franceshi focused on retention of the spherical shape and migration of the humeral head [10]. They classified as defined on two criteria: the sphericity and upward migration of the humeral head into 3 types, ascending form, centered form, and destructive form, in 55 shoulders from 44 RA patients and evaluated the rotator cuff status and improved range of motion after total shoulder arthroplasty (TSA) or humeral head replacement (HHR). Sirveaux et al. classified four types of glenoid erosion associated with cuff tear arthropathy. In type E0, the head of the humerus migrated upwards without erosion of glenoid. Type E1 was defined by a concentric erosion of the glenoid. In type E2, there was an erosion of the superior part of glenoid and in type E3 the erosion extended to the inferior part of the glenoid [7]. They investigated distribution and size of the scapular notch according to the type of glenoid. Compared with this study in patients with RA shoulder, E0 is similar Type I, E1 is Type II, and E2/3 is Type III. Although, there are few shoulders (6 of 48 shoulders in Type III) of E2 in RA shoulder, we decided E2 and E3 in Favard's classification as Type III in this study. Our study focused mainly on the morphology of the glenoid fossa. We classified the characteristics into 3 types with the objective of creating a simple classification that could be used by physicians who are unfamiliar with radiological evaluation of the shoulder joint. While creating the classification, we recognized that there are differences in each type depending on whether there is upward migration of the humeral head. Considering previous studies on the significant influence of the postoperative results of TSA or HHR and the role of rotator cuff function [13], we added the classification of upward migration of the humeral head (initially proposed by Oizumi et al.) as a simple indictor to evaluate rotator cuff function, and thereby developed a new classification. 

In cases involving both shoulders, most shoulders belonged to the same type. Factors distinguishing the status of the patient were therefore assumed to play a role in each type. Since Type III was common with cane or wheelchair use, the Steinbrocker functional class, and a history of leg surgery, there was a probable relationship of the magnitude of weight bearing on the arms and the type of glenoid deformity. Type I-N and II-N tended to display a greater range of motion (flexion, external rotation) and lower incidence of rotator cuff problems. The fact that Type I changed to II or III, Type II changed to III, and Type N changed to U in cases of patients who were observed during the course of the study indicated the possibility that a decrease in the range of motion and progressive damage to the rotator cuff are related to the advance from Type I to II or III and from Type N to U.

Many cases of Type III-U showed upward migration of the humeral head, thereby indicating a relationship with rotator cuff tear. Rotator cuff tears could only be evaluated, however, in patients who had undergone an MRI or from the surgical findings in patients who had undergone surgery. No significant difference could be detected, perhaps because of the small number of cases.

We plan to conduct further studies evaluating the relationship between each classification type and the surgical results and differences observed after assessing the A-P X-ray scans and performing three-dimensional evaluation using CT/MRI.

## 5. Conclusion

There are three types of characteristic changes in shape of the glenoid fossa using radiological examinations of the RA shoulder. We were able to classify a total of six types of deformities by adding the problem of upward migration of the humeral head. 

Both shoulders were of the same type in many cases. Factors distinguishing the patient in determining the classification appeared to be use of a cane or wheelchair, Steinbrocker functional class, and leg surgery.

Many cases changed from Type I to II or III during the course of observation, and a classification to evaluate grading of the advance of joint destruction appeared to be possible as well.

## Figures and Tables

**Table 1:** Classification of functional capacity in rheumatoid arthritis Steinbrocker functional classification.

Class I	Complete functional capacity with ability to carry on all usual duties without handicaps
Class II	Functional capacity adequate to conduct normal activities despite handicap of discomfort or limited mobility of one or more joints
Class III	Functional capacity adequate to perform only few or none of the duties of usual occupation or of self-care
Class IV	Largely or wholly incapacitated with patient bedridden or confined to wheelchair, permitting little or no self-care

**Table 2:** Numbers of shoulders and average age into each types.

	Shoulders			Average age (years old)
		sex		
		Men	Women	
Type I N	36	5	31	60.6 (33~77)
Type I U	9	4	5	67.3 (55~75)
Type II N	57	12	45	61.7 (21~80)
Type II U	0	0	0	—
Type III N	16	2	14	62.5 (25–81)
Type III U	32	8	24	61.8 (50~78)

**Table 3:** The relationship between this particular classification and certain patient characteristic.

Factors	Significant difference (Type)	*P* value
Age	—	NS

Morbidity time	—	NS

Use of canes	III U > II N	0.002

Use of a wheelchair	III U > I (U, N)	0.025, 0.0029
	III U > II N	0.0005

Class	III U > I N	<0.0001
	III U > II N	0.0003

Past history of operation (the lower limbs)	III N > II N	0.0566 (NS)
	III U > II N	0.0622 (NS)

ROM flexion	I N > I U, III U	0.0024, 0.0002
	II N > I U, III U	0.0247, 0.0088

ER/IR	II N > III U/—	0.0384/NS

Existence of cuff tear	I U > I N	0.0118
	I U > II N	0.0047

ANOVA Fisher's PLSD test.
